# Performance of the ^13^C-urea breath test for the diagnosis of *H. pylori* infection using a substrate synthesized in Brazil: A preliminary study

**DOI:** 10.6061/clinics/2018/e16-553

**Published:** 2018-05-28

**Authors:** Luiz Gonzaga Coelho, Carlos Roberto Sant'Ana, Ricardo Brandt de Oliveira, Raíra César e Cezar, Aline Cordeiro Campos de Araujo, Raisa Cristina Teodoro da Silva, Osmar Reni Trindade, Maria Clara Coelho, Eduardo Ferrioli, José Albertino Bendassolli

**Affiliations:** I Instituto Alfa de Gastroenterologia, Hospital das Clinicas, Universidade Federal de Minas Gerais, Belo Horizonte, MG, BR; II Centro de Energia Nuclear na Agricultura (CENA/USP), Universidade de Sao Paulo, Piracicaba, SP, BR; III Departamento de Clinica Medica, Faculdade de Medicina de Ribeirao Preto, Universidade de Sao Paulo, Ribeirao Preto, SP, BR

**Keywords:** ^13^C-urea breath test, ^13^C-urea, *Helicobacter pylori*, *H. pylori* diagnosis

## Abstract

**OBJECTIVE:**

The ^13^C-urea breath test is the main non-invasive test for the diagnosis of *Helicobacter pylori* infection. The availability of this test throughout the country is limited, mainly due to the difficulty in obtaining the labeled isotope from abroad. Recently, researchers from the Nuclear Energy Center in Agriculture at the University of São Paulo (CENA/USP) succeeded in synthesizing ^13^C-enriched urea for *Helicobacter pylori* diagnosis. The aim of the study was to compare the performance of the ^13^C-urea breath test using ^13^C-urea acquired abroad with that of a test using ^13^C-urea synthesized in Brazil.

**METHOD:**

Sixty-four dyspeptic patients participated in the study (24 men and 40 women). Initially, the patients performed the ^13^C-urea breath test using the imported substrate (Euriso-Top, France). Seven to fourteen days later, all the patients repeated the test using the Brazilian substrate. The samples from both examinations were processed in an infrared isotope analyzer (IRIS, Wagner Analisen Technik, Germany), and all delta over baseline (DOB) [%] values above four were considered positive results.

**RESULTS:**

Twenty-seven patients (42%) exhibited negative results for *Helicobacter pylori* infection, and thirty-seven patients (58%) exhibited positive results when tested using the foreign substrate (gold standard). There was a 100% concordance regarding the presence or absence of infection when the gold standard results were compared with those obtained using the Brazilian substrate.

**CONCLUSIONS:**

Similar performance in the diagnosis of *Helicobacter pylori* infection was demonstrated when using the ^13^C-urea breath test with the Brazilian ^13^C-urea substrate and the test with the substrate produced abroad. This validation represents an important step toward increasing the availability of the ^13^C-urea breath test throughout the country, which will have a positive influence on the management of *Helicobacter pylori* infection.

## INTRODUCTION

*Helicobacter pylori* (*H. pylori*) was first identified by Marshall and Warren 1 in 1983 in Australia and has since been found all over the world, in at least 50% of the world’s population. *H. pylori* is the major acquired environmental factor in the pathogenesis of a large spectrum of diseases, such as acute and chronic gastritis, gastric and duodenal ulcers, gastric carcinomas and mucosa-associated lymphoid tissue (MALT) lymphomas 2.

*H. pylori* can be detected in the gastric mucosa by different methods, including detection in fragments that are removed during endoscopy (invasive tests), which can be used for histopathological, microbiological, colorimetric (urease test) and molecular studies, and nonendoscopic techniques (non-invasive tests), including searching for *H. pylori* with breath tests that use carbon 13-labeled urea, which are based on the organism’s ability to produce high quantities of the urea enzyme. The principle of the latter test is based on the ability of *H. pylori* (if present in the gastric setting) to breakdown orally absorbed ^13^C urea into CO_2_. The ^13^CO_2_ diffuses into the blood and is excreted via the lungs; therefore, it can be easily measured in the exhaled air using a mass or infrared spectrometer 3. Initially described in 1987 4, the urea breath test (UBT) is considered the most well-known, well-standardized and widely used breath test. This test is non-radioactive, reproducible and safe and can be performed in children and pregnant women. Multiple studies and a meta-analysis have demonstrated that the sensitivity and specificity of this test are greater than 95% compared with the results of serology and other diagnostic methods 5-7.

This test has been validated and performed since 1998 in the adult population of Belo Horizonte City for initial diagnosis of *H. pylori* infection and/or post-treatment assessment 8. However, the use of this test throughout the country remains unacceptably restricted to a few university centers 8-11. One of the main barriers regarding the use of this test is the difficulty in obtaining the labeled isotope, as the technology for carbon separation (i.e., ^13^C and ^12^C) has not been transferred by the countries that developed it due to economic reasons and strategic interests. Recently, researchers from the Center of Nuclear Energy in Agriculture of the University of São Paulo (CENA/USP) were able to achieve the synthesis of urea (^13^CO(NH_2_)_2_) with 99% ^13^C atoms with a competitive production process and a reasonable cost compared with the international market value 12.

The aim of this study was to compare the performance of the UBT using ^13^C-urea that was acquired abroad with that of the test using ^13^C-urea that was synthesized in Brazil in the same population.

## PATIENTS AND METHODS

The study was conducted at the Breath Tests Laboratory of the Alfa Institute of Gastroenterology at the University Hospital of the Federal University of Minas Gerais, Belo Horizonte, MG, Brazil.

### Patients

The patients were recruited from those who were routinely sent to undergo a UBT at the Breath Tests Laboratory for the diagnosis of *H. pylori*. The patients received a full explanation of the objectives of the study, and once they agreed to participate in the study, they provided written informed consent. For inclusion in the study, the patients must not have used proton pump inhibitors (PPIs) in the preceding 15 days or antibiotics in the preceding 30 days because these medications could interfere with the UBT results.

### ^13^C-urea breath test

All the participants initially underwent a UBT using ^13^C-urea acquired abroad (Euriso-top, France); subsequently, they underwent another UBT using the Brazilian substrate (CENA/USP) within 7-14 days. The technical aspects of the test are briefly described as follows. After an overnight fast, an initial breath sample was collected from the patients by having them blow into a balloon to determine the basal test results. Next, the patients were administered 75 mg of ^13^C-urea added to a glass (200 ml) of pure orange juice. Thirty minutes later, an additional collection of expired air was performed, and the material was analyzed using an infrared analyzer (IRIS, Wagner Analysen-Technik, Germany). The results were obtained on on a delta basis per absolute mil or as DOB “*delta over baseline*”, which indicates the change in the ^13^CO_2_/^12^CO_2_ ratio from the metabolic activity induced by administering the labeled urea. DOB values above 4% were considered positive on the UBT, as previously validated by our group 8.

### Statistics

Statistical analyses were performed using descriptive statistical techniques (tables and percentages). To compare the pairs of samples, we used the non-parametric Wilcoxon signed-rank test. The analyses were conducted using Minitab 16, and the level of significance was set at *p*<0.05.

## RESULTS

Sixty-four dyspeptic patients completed the study (24 men and 40 women, mean age 53 years [min. 25, max. 85]). Overall, 27/64 (42%) patients tested negative for *H. pylori*, and 37/64 (58%) of the patients tested positive according to the UBT using the imported substrate (i.e., the gold standard).

### Comparison of the UBTs using the imported and Brazilian ^13^C-urea

**Figure 1:**
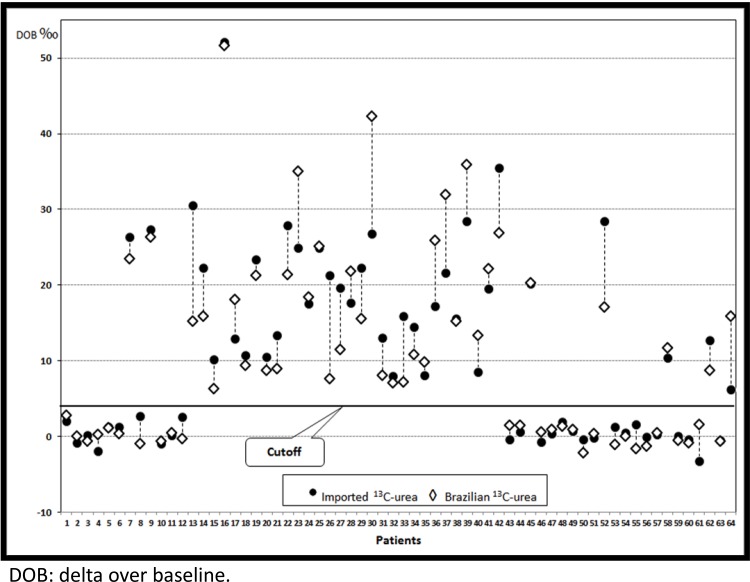
^13^C-urea breath test values from 64 patients who performed the test using ^13^C-urea acquired abroad and ^13^C-urea synthesized in Brazil.

**Figure 2:**
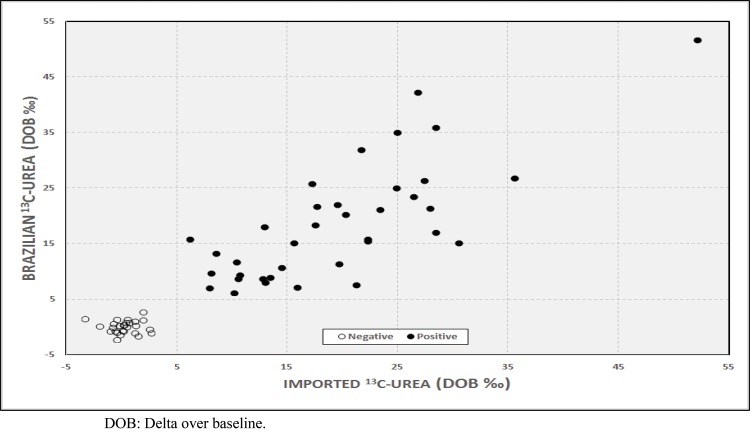
Scatterplot of the DOB values based on imported ^13^C-urea *vs* those based on Brazilian ^13^C-urea.

Figure 1 presents the results of the analyses for all 64 patients based on the cut-off value. One hundred percent concordance regarding positivity and negativity for infection resulted when comparing the results based on the imported and Brazilian substrates. A Wilcoxon test revealed that there were no significant differences between the two methods with regard to the differences observed between the positive and negative results based on the Brazilian substrate, and the DOB values based on the Brazilian substrate were not underestimated or overestimated compared with the values based on the imported urea (*p*=0.23). Table 1 presents the DOB values obtained from infected and uninfected patients using both tests, and Figure 2 presents a scatterplot of the 128 DOB values obtained.

## DISCUSSION

Our results clearly demonstrated that the ^13^C-urea substrate synthesized by CENA/USP in Brazil could achieve the same performance on the UBT for the diagnosis of *H. pylori* infection as the substrate produced by foreign biotechnology companies; this finding could facilitate the dissemination of this test throughout Brazil.

The UBT is now considered the gold standard non-invasive test for detecting the presence or absence of *H. pylori* infection 5. This test is superior to serology because the decline in antibody titers after *H. pylori* eradication is slow and unpredictable 13,14. Although stool antigen tests can achieve similar sensitivity and specificity with the use of monoclonal antibody-based ELISA, they are considered less acceptable and practical than UBT in daily practice 5. The UBT is particularly suitable in all clinical conditions in which endoscopy is not strictly necessary and for verifying the success of eradication regimens 7. More recently, the UBT, instead of prompt endoscopy, has been considered the main test for use in the context of test-and-treat strategy for the management of young people with uninvestigated dyspepsia from regions with a high *H. pylori* prevalence, such as Brazil 5,15.

Unfortunately, the UBT has not yet been incorporated into daily gastroenterological practice in Brazil, and its availability remains restricted to large cities and university centers 8-11. To enhance the availability of this test in Brazil, numerous challenges must be addressed, including difficulties in acquiring infrared spectrometers, the lack of regular reimbursement by health care systems, the poor familiarity of general physicians with the UBT, and problems related to the acquisition of ^13^C-urea from abroad.

This study is a preliminary report that examined the performance of a small volume of ^13^C-urea manufactured in Brazil for the first time. Further studies involving all ethical and regulatory aspects related to the development of a new drug should be performed to achieve registration with the Brazilian Health Regulatory Agency (Anvisa) before this test is entered into the market. The results described here should stimulate the development of large-scale production of ^13^C-urea to facilitate the broader use of the UBT, and the subsequent technological production advances should stimulate a dramatic reduction in the cost of the substrate, which is currently estimated at approximately US $15.00 per test 12.

In conclusion, the ^13^C-urea substrate synthesized at a Brazilian university center exhibited a performance similar to that of the substrate produced by a foreign company when used in the UBT to diagnose the presence or absence of *H. pylori* infection. This validation represents an important step toward improving the availability of the UBT throughout the country, which will positively influence the diagnosis and therapeutic management of *H. pylori* infection.

## AUTHOR CONTRIBUTIONS

Coelho LG, Oliveira RB and Ferrioli E conceived and designed the study. Cezar RC, Araujo AC, Silva RC and Trindade OR were responsible for the data collection and processing of the samples. Bendassolli JA and Sant’Ana CR produced the substrate for the study. Coelho LG, Cezar RC and Coelho MC were involved in qualitative data analysis and manuscript writing.

## Figures and Tables

**Table 1:** DOB values (^13^C-imported urea and ^13^C-Brazilian urea) of the positive and negative patients.

Descriptive statistics	Groups			
	Uninfected patients (n=27)		Infected patients (n=37)	
	^13^C-Imported urea	^13^C-Brazilian urea	^13^C-Imported urea	^13^C-Brazilian urea
**Min**	-3.30	-2.20	6.15	6.25
**Mean**	0.24	0.09	19.59	18.68
**Standard deviation**	1.32	1.12	9.21	10.57
**Max**	2.65	2.70	52.05	51.65

DOB: Delta over baseline.
